# Supplementary material for: Asynchronous magnetic resonance elastography: Shear wave speed reconstruction using noise correlation of incoherent waves
Source: Magn Reson Med. 2022 Oct 27;89(3):990–1001. doi: 10.1002/mrm.29502 (PMC9792433; doi:10.1002/mrm.29502)
Supplement: Supplementary file 1 — DATA S1 MATLAB code used to generate the numerical simulations of Figure 1 [file MRM-89-990-s002.zip › k-Wave/helpfiles/powerLawKramersKronig.html]

powerLawKramersKronig :: Functions (k-Wave)


# powerLawKramersKronig

Calculate dispersion for power law absorption.

## Syntax

```
c_kk = powerLawKramersKronig(w, w0, c0, a0, y)
```

## Description

`powerLawKramersKronig` computes the variation in sound speed for an attenuating medium using the Kramers-Kronig for power law attenuation where `att = a0*w^y`. The power law parameters must be in Nepers / m, with the frequency in rad/s. The variation is given about the sound speed `c0` at a reference frequency `w0`.

## Inputs

|  |  |
| --- | --- |
| `w` | input frequency array [rad/s] |
| `w0` | reference frequency [rad/s] |
| `c0` | sound speed at `w0` [m/s] |
| `a0` | power law coefficient [Nepers/((rad/s)^y m)] |
| `y` | power law exponent, where 0 < `y` < 3 |

## Outputs

|  |  |
| --- | --- |
| `c_kk` | variation of sound speed with `w` [m/s] |

## Examples

- Modelling Power Law Absorption

## See Also

`db2neper`
